# Transporter Associated With Antigen Processing (TAP) 1 Gene Polymorphisms and Risks of Urothelial Cell Carcinoma Among the Japanese Population

**DOI:** 10.7759/cureus.52310

**Published:** 2024-01-15

**Authors:** Khine Zin Aung, Sa Tin Myo Hlaing, Putri Damayanti, Tamanna Tabassum, Hiromasa Tsukino, Takuji Hinoura, Yoshiki Kuroda

**Affiliations:** 1 Department of Biostatistics, University of Kentucky College of Medicine, Lexington, USA; 2 Department of Public Health, Faculty of Medicine, University of Miyazaki, Miyazaki, JPN; 3 Department of Urology, Junwakai Memorial Hospital, Miyazaki, JPN

**Keywords:** urology, tap1 gene, case-control, carcinogenesis, cancer risk

## Abstract

Urothelial cell carcinoma is one of the costliest types of cancer because of its recurrence, lengthy course of therapy, and tendency to lead to further complications. Gene polymorphisms are one of many factors that are thought to cause the carcinogenesis of urothelial cell carcinoma. Two single-nucleotide polymorphisms (SNPs) of the transporter associated with antigen processing (TAP) 1 gene and their relationship with the risks of urothelial cell carcinoma in the Japanese population were examined in this study by using polymerase chain reaction (PCR), restriction fragment length polymorphism (RFLP) for genotyping and statistical analysis.

The adjusted odd ratios with 95% confidence interval (CI) of the mutant types (A/G+G/G) in females for the I333V and D637G polymorphisms are 2.28 (1.11-4.66) and 2.50 (1.21-5.17), respectively. The findings showed that females with the (A/G+G/G) genotype are more likely to develop urothelial cell carcinoma than those with the A/A genotype. Any correlation between smoking and gene polymorphism was absent. Results indicate that TAP1 gene polymorphisms and the risk of urothelial cell carcinoma are related in females.

## Introduction

Urothelial cell carcinoma, which comprises upper tract urothelial cell carcinoma (5-10% of urothelial cell carcinoma) and bladder carcinoma (more than 90% incidence of all urothelial malignancies), is one of the most common types of cancer [1]. The recurrence rate of urothelial cell carcinoma could appear to be high, and the prognosis is poor. The prevalence rate of urothelial cell carcinoma is higher among males compared to females [2]. According to National Cancer Center Japan statistics in 2014, the five-year survival rate for patients diagnosed with bladder cancer from 2010 to 2011 was 68.4%, and the five-year survival rate for overall renal pelvis and ureter cancer was 49% [3]. Recently, incidences of urothelial cell carcinoma have either moderately increased or remained high in most developed countries [4]. On the other hand, there have been improvements in the diagnosis and treatment of urothelial cell carcinoma, as well as a greater awareness of important risk factors such as cigarette smoking and occupational chemical exposure [5]. The carcinogenesis of urothelial cell carcinoma is thought to be multifactorial, and many factors, such as genetic factors and environmental factors, were included as modulators of urothelial cell carcinoma, including the polymorphisms of genes among several metabolic enzymes [6-8].

Major histocompatibility complex class 1 (MHC-1) molecules play a key role in the immune surveillance of infected and transformed cells, which bind the intracellularly processed peptides and travel to the cell surfaces, where they present peptide fragments to cytotoxic T lymphocytes (CTL). In the process of antigen presentation, the peptide fragments digested by proteosomes in the cytoplasm of a cell are transported to the endoplasmic reticulum by the transporter associated with an antigen-processing protein complex (TAP). As a TAP, there are heterodimer integral membrane proteins of both TAP1 and TAP2 that provide a supply of processed peptides to MHC1 [9]. The newly formed MHC-1 peptide complex shifts to the cell surface through the Golgi apparatus. The T cell receptor (TCR) and co-receptor of CD8 recognize the MHC1 complex, and the T cell interaction begins. The polymorphisms of TAP genes may change the structure and function of the complex and induce profound effects on immune surveillance and cancer susceptibility [10].

The TAP1 gene has seven alleles, and TAP1 polymorphisms are linked to various types of cancer carcinogenesis, such as protective risk or precipitating risk [10]. However, there has not yet been any research on TAP1 polymorphism and urothelial cell carcinoma risk carried out on the Japanese population that I know of. Therefore, we decided to investigate the association between urothelial cell carcinoma and two polymorphisms of the TAP1 gene (I333V and D637G). Since smoking was known to be related to the carcinogenesis of several cancers, and because gender can also influence carcinogenesis, we analyzed the correlation between each gene polymorphism and cancer susceptibility, taking into account both smoking habits and gender.

## Materials and methods

Study population

A total of 266 patients with urothelial cell carcinoma (cases group) and 263 healthy subjects (control group), recruited from the Japanese population between September 1992 and December 2006, were included in this research. Patients were diagnosed at the University of Occupational and Environmental Health (UOEH) Hospital and the University of Miyazaki Hospital in Japan, and all diagnoses were confirmed through histopathologic examinations according to World Health Organization classification. We collected information from participants' medical histories or hospital records. We included controls who did not have malignant conditions and our criteria for selection are controls who are free from exposure to carcinogens, radiations, and toxic materials.

The necessary personal information of both the cases and control groups, such as occupation, smoking history, residence, and history of illness, was obtained by a self-administered questionnaire. Any participant who had been exposed to carcinogens, heavy metals, or radiation in their surrounding environment was excluded from either group. We divided the subjects into two groups: “smokers” included current or former smokers, while “non-smokers” were classified as people who had never smoked in their lifetime. All participants were informed of the general process of the research and gave written informed consent. This study was conducted in accordance with the Declaration of Helsinki and was approved by the ethical committee of the University of Miyazaki (approval number: 239).

The genotyping of two gene polymorphisms of TAP

Genomic DNA was extracted from a peripheral blood lymphocyte by conventional methods [11]. A restriction fragment length polymorphism (PCR-RFLP) technique was performed to detect two single-nucleotide polymorphisms (SNPs), such as the I333V and D637G of the TAP1 gene. The forward and reverse primers of first-step polymerase chain reaction (PCR), forward and reverse primers, PCR product, and restriction enzymes are indicated in Table 1. The conditions of PCR for both codons 333 and 637 were as previously described [12].

**Table 1:** Primers of PCR, PCR products, restriction enzymes and digested fragments PCR: polymerase chain reaction

Variant	Primers	PCR product	Restriction enzyme	Digested fragments	
I333V	F: GCAGGTAACATC ATGTCTCG	430bp	Bcl1	(A/A :274bp,156bp), (A/G :430bp,274bp,156bp), (G/G :430bp)	
	R: GACAGATTGTGGGGAGAAGC				
D637G	F: CAGTAGTCTTGCCTTTATCC	405bp	Acc1	(A/A :260bp,145bp), (A/G :405bp,260bp,145bp), (G/G :405bp)	
	R: ATGACTGCCTCACCTGTAAC				

Each PCR product was digested by restriction enzymes. The digested fragments were then analyzed by electrophoresis on 3% agarose gel staining ethidium bromide and visualized in UV light. Table 1 shows the digested fragments of the PCR products.

Statistical analysis

All statistical analyses were performed using R version 4.1.1 (The R Foundation for Statistical Computing, Vienna, Austria). For categorical variables, Pearson’s chi-square test was used for analysis. The calculation of the Hardy-Weinberg equilibrium was also performed using the x2 test. The t-test was applied for two categorical numerical analyses. To reduce the effects of confounding factors, gender, age and smoking were adjusted using a logistic regression analysis with multivariate and odds ratio (OR) and a 95% confidence level (95% CI) are calculated. Furthermore, we stratified the analysis according to gender and smoking habits (smoking and non-smoking groups). We then analyzed the results a second time. In all statistical analyses, any P value which is less than 0.05 is considered to be statistically significant.

## Results

The general characteristics of 266 urothelial cell carcinoma patients (216 males and 50 females) and 263 control individuals (126 males and 137 females) are shown in Table 2. Significant associations were detected between cases and control groups with regard to both gender and the smoking rate. We found that 18.79% (50/266) of cases and 52.09% (137/263) of controls were female. In addition, the distribution of smokers in the case subjects (72.56%: 193/266) was significantly higher than that of controls (50.57%: 133/263). However, there was no significant association with the average Brinkman index between the cases and the controls. The mean age of cases and controls were not significantly different (69.3±10.8 V.S. 68.8±12.3).

**Table 2:** The base characteristics of cases and controls ** P<0.01, SD: standard deviation

Characteristics	Case (Male/Female)	Control (Male/Female)
Number of participants	266 (216/50)	263 (126/137**)
Age (mean±SD)	69.3±10.8 (69.4±10.8/69.2±10.8)	68.8±12.3 (67.7±12.3/69.9±12.3)
Number of never-smokers	73 (33/40)	130 (22/108)
Number of current or ever-smokers	193 (183/10)	133** (104/29)
Brinkman Index	809.0±440.0 (822.0±439.4/493.1±335.9)	749.2±515.39 (846.0±502.4/401.9±408.1)

The distribution of TAP1 polymorphisms such as I333V and D637V between the cases and control groups is shown in Table 3. The genotypes of the control group among two polymorphisms were measured on a Hardy-Weinberg equilibrium (P value >0.05). The only G/G genotypes of I333V polymorphism were found to be significant in the cases when compared to controls when adjusted by age, gender, and smoking habits (Table 3).

**Table 3:** The relation between urothelial cancer and the genotypes of TAP1 (I333V, D637G) polymorphisms Adjusted by age, gender, smoking status, * P<0.05 TAP1: transporter associated with antigen processing 1 gene

Variant	Genotype	Case	Control	Crude OR	Adjusted OR
I333V	A/A	197	209	ref	ref
	A/G	62	53	1.24 (0.82-1.88)	1.32 (0.85-2.07)
	G/G	7	1	7.43 (0.91-60.91)	9.79 (1.10-87.00)*
	A/G+G/G	69	54	1.36 (0.90-2.03)	1.46 (0.95-2.27)
D637G	A/A	201	212	ref	ref
	A/G	59	50	1.25 (0.82-1.90)	1.36 (0.86-2.15)
	G/G	6	1	6.33 (0.76-53.03)	6.88 (0.75-62.96)
	A/G+G/G	65	51	1.34 (0.89-2.03)	1.47 (0.94-2.30)

We evaluated the relation between cases and controls concerning the two polymorphisms’ distribution separated by gender (Tables 4-5). Among females, A/G+G/G genotypes of the I333V polymorphism were predominant in cases comparing controls (Table 5). Regarding D637G polymorphisms in females, A/G and A/G+G/G genotypes were predominant in cases comparing controls in the same manner as the I333V.

**Table 4:** The relation between urothelial cancer and the genotypes of TAP1 (I333V, D637G) polymorphisms among males Adjusted by age and smoking status, OR: odd ratio, ** P<0.01, * P<0.05 TAP1: transporter associated with antigen processing 1 gene

Variant	Genotype	Case	Control	Crude OR	Adjusted OR
I333V	A/A	165	99	ref	ref
	A/G	46	27	1.02 (0.60-1.75)	1.02 (0.59-1.75)
	G/G	5	0	ー	ー
	A/G+G/G	51	27	1.13 (0.67-1.92)	1.13 (0.66-1.93)
D637G	A/A	169	100	ref	ref
	A/G	42	26	0.96 (0.55-1.65)	0.96 (0.55-1.66)
	G/G	5	0	ー	ー
	A/G+G/G	47	26	1.07 (0.62-1.83)	1.07 (0.66-2.15)

**Table 5:** The relation between urothelial cancer and the genotypes of TAP1 (I333V, D637G) polymorphisms among females Adjusted by age and smoking status, OR: odd ratio, ** P<0.01, * P<0.05 TAP1: transporter associated with antigen processing 1 gene

Variant	Genotype	Case	Control	Crude OR	Adjusted OR
I333V	A/A	32	110	ref	ref
	A/G	16	26	2.12 (1.01-4.42)*	2.09 (1.00-4.38)
	G/G	2	1	6.88 (0.60-78.29)	7.03 (0.61-80.99)
	A/G+G/G	18	27	2.29 (1.12-4.68)*	2.28 (1.11-4.66)*
D637G	A/A	32	112	ref	ref
	A/G	17	24	2.48 (1.19-5.17)*	2.45 (1.17-5.14)*
	G/G	1	1	3.50 (0.21-57.53)	3.89 (0.21-60.15)
	A/G+G/G	18	25	2.52 (1.22-5.19)	2.50 (1.21-5.17)*

We also evaluated the relationship between genotypes among two polymorphisms separated by smoking habits (smoking vs. non-smoking). These are described in Tables 6-7. However, we could not provide any interaction with genotypes of D637G and smoking.

**Table 6:** The relation between urothelial cancer and the genotypes of TAP1 (I333V, D637G) polymorphisms in smokers Adjusted by age and gender, OR: odd ratio, ** P<0.01, * P<0.05 TAP1: transporter associated with antigen processing 1 gene

Variant	Genotype	Case	Control	Crude OR	Adjusted OR
I333V	A/A	148	108	ref	ref
	A/G	40	25	1.17 (0.67-2.04)	1.22 (0.68-2.17)
	G/G	5	0	ー	ー
	A/G+G/G	45	25	1.31 (0.76-2.27)	1.36 (0.77-2.41)
D637G	A/A	151	109	ref	ref
	A/G	37	24	1.11 (0.63-1.97)	1.14 (0.63-2.06)
	G/G	5	0	ー	ー
	A/G+G/G	42	24	1.26 (0.72-2.21)	1.29 (0.72-2.30)

**Table 7:** The relation between urothelial cancer and the genotypes of TAP1 (I333V, D637G) polymorphisms in non-smokers Adjusted by age and gender, OR: odd ratio, ** P<0.01, * P<0.05 TAP1: transporter associated with antigen processing 1 gene

Variant	Genotype	Case	Control	Crude OR	Adjusted OR
I333V	A/A	49	101	ref	ref
	A/G	22	28	1.62 (0.84-3.12)	1.50 (0.75-3.01)
	G/G	2	1	4.12 (0.36-46.57)	5.94 (0.52-68.17)
	A/G+G/G	24	29	1.71 (0.90-3.23)	1.64 (0.83-3.21)
D637G	A/A	103	50	ref	ref
	A/G	26	22	0.57 (0.30-1.11)	0.58 (0.29-1.16)
	G/G	1	1	0.49 (0.03-7.92)	0.35 (0.02-5.82)
	A/G+G/G	27	23	0.57 (0.30-1.09)	0.56 (0.28-1.12)

## Discussion

In the complex journey to carcinogenesis, MHC-I plays an important role in the immune surveillance system for abnormal and degraded cells [13]. In the process of antigen presentation and processing, transporter proteins (TAP1 and TAP2) are major components of antigen processing machinery (APM) located in the human leukocyte antigen (HLA) class 2 region and are responsible for the translocation of peptides, which were produced from the proteosomes, from the cytosol to the lumen of the endoplasmic reticulum [14].

In cases of abnormalities in the TAP gene, tumor cells escape CTL recognition and lead to carcinogenesis and further progress of carcinogenesis. Changes in the structures or functions in TAP lead to disruptions in APM by MHC and create an impact on the immune escape and downregulation of the MHC complex, leading to the heightened possibility of tumor cell invasion and subsequently leading to a reduced prognosis for CD8-targeted cancer treatment [15].

In addition, translocation of digested peptides from the cytosol to the endoplasmic reticulum is carried out by adenosine triphosphate (ATP) binding and hydrolysis, as the TAP gene possesses domains of the ABC transporter family that are involved in ATP binding and hydrolysis [16]. The ATP-binding cassette (ABC) transporters are also responsible for the translocation of substrates such as amino acids, ions, and sugars across membranes and are involved in maintaining genetic stability [17]. Therefore, there is a possibility that changes in the TAP gene (which belongs to the ABC transporter superfamily) may contribute to genetic events such as DNA repair in carcinogenesis. In addition, an effective immune surveillance system is dependent on the antigen presentation by the TAP gene. Functional changes in the TAP gene are due mostly to mutations [13,14], impaired expression, and increased degradation [14]. However, the detailed functional changes of individuals possessing SNPs of the TAP gene have not yet been widely evaluated.

Table 3 indicates that the G/G genotype of the I333V polymorphism was predominant among the cases, which further indicates that this genotype could be a risk factor for urothelial cell carcinoma.

Ozbas-Gerceker et al. reported that the G/G genotype appeared to be a risk for multiple myeloma [18], and our results were consistent with theirs. On the other hand, a study of nasopharyngeal carcinoma with the polymorphisms of I333V and codon 637 (D637G) among a group of Tunisian patients showed that the A allele could be a significantly increased risk factor for carcinogenesis of nasopharyngeal carcinoma [19], while Suryanarayana et al. claimed that the G allele of I333V polymorphism acted as a protective factor against HPV associated oropharyngeal cancer [20]. In short, the influence of I333V polymorphism has been controversial concerning its relation to carcinogenesis.

We evaluated the relationship between polymorphism and cancer risk by stratifying gender as shown in Tables 4-5, with the A/G+G/G genotype of I333V polymorphism being significantly predominant in the cases when compared to A/A genotypes, particularly in females. Similarly, the A/G and A/G+G/G genotypes of D637G polymorphism were significantly predominant in the cases. However, we could not detect any relationship between the two polymorphisms and urothelial cell carcinoma in males. In both polymorphisms, since the number of cases and control of the G/G genotype were few, it was thought that we could not evaluate the effects of the G/G genotype. Therefore, we cannot say that the G allele of both polymorphisms could be a risk factor for urothelial cell carcinoma. However, Zou et al. have reported that carriers of the A/G genotype of D637G have higher risks of having esophageal cancer than other genotypes [12]. A further study of nasopharyngeal carcinoma in the Han Chinese population also revealed that homozygous G/G and heterozygous A/G carriers of D637G polymorphism were found to be more susceptible to nasopharyngeal cancer when compared with other genotypes [21]. Our findings thus seem to be consistent with the previous study. However, Jia Yang et al. reported that the G allele of D637G polymorphism was associated with a reduced risk of cervical cancer, while Elham et al. reported that the A allele contributed to a higher risk of nasopharyngeal cancer. The influence of the D637G polymorphism was controversial concerning carcinogenesis in a manner similar to the I333V polymorphism.

According to our findings, TAP1 polymorphisms (I333V and D637G) could be a risk factor in females but not in males. Concerning the interaction between gene polymorphisms and gender, this study was found to be in contrast with some previously reported studies. It has been reported that TAP1 polymorphism has increased susceptibility in both genders in colon cancers [11]. For example, Holipah et al. claimed that mutant type (G/G) of PER1(rs 3027188) is protective against colon cancer in females [22], and also indicated that mutant T/T genotype of PER3 rs2640908 became a protective factor against colon cancer in males [23]. However, Dresler CM et al. reported that the polymorphisms of cytochrome P450 1A1 (CYP1A1) (exon 7) were a risk factor for lung cancer, particularly in females [24]. They further pointed out that the different effects of polymorphism by gender were derived from the influence of female sex steroid hormones such as estrogen.

In general, however, the incidence of urothelial cell carcinoma is predominant in males [2], while urothelial cell carcinoma in females tends to be more invasive and recurrent [25]. Some studies have suggested that estrogen receptor ERα and ERβ expressions were different depending on the kind of cancer [26], and that there was higher expression of these in urothelial cell carcinoma [27], while the androgen receptors are expressed in both normal and tumor bladder epitheliums equally regardless of gender [28]. Some studies found that androgen deprivation by androgen receptor modulation impacted the prognosis and treatment of bladder cancer both in vivo and in vitro [29]. Other reports have indicated that androgen receptor deprivation improved urothelial cell carcinoma recurrence from pathways other than those of ERα and ERβ [26]. Our study concerning carcinogenesis between polymorphism and urothelial cancer risk stratified by gender could also be related to the sex hormone receptor’s function. In short, further studies need to clarify the relationship between gender and genotypes and their impact on carcinogenesis.

We didn’t detect any interaction between genotype and smoking habits (Tables 6-7). Several reports have indicated the interaction between some polymorphism and smoking [30], and other studies failed to detect the association [22,23]. Therefore, the interaction between smoking and genotype also indicates discrepancies due to the complexity of gene-environment interactions, variation in smoking habits, and various study methodologies.

There were some limitations in our study. We were unable to include data about alcohol consumption, diet, details of smoking history, exercise, and body mass index (BMI). Our study also did not assess the tumor stage, location, pathological type, and prognosis. Furthermore, since we were required to adopt the hospital controls, we could not rule out the possibility that our controls contained some kind of bias. However, there have been few studies conducted concerning the relation between TAP1 polymorphism and carcinogenesis. Our study is one of these few, and, to the best of our knowledge is the first report on TAP1 polymorphisms (I333V and D637G) with urothelial cell carcinoma risks in the Japanese population.

## Conclusions

As a conclusion, according to our study, the G allele of TAP1 polymorphisms (I333V and D637G) was susceptible to urothelial cell carcinoma in females, as we detected some interactions between TAP1 polymorphisms (I333V and D637G) and gender. However, we did not detect any interaction with smoking. Since the relationship between polymorphism and gender is controversial, further research will be needed. However, this is the first study conducting research on the relationship between TAP1 polymorphisms (I333V and D637G), and we hope our results can contribute to the development of further research on the relationship between TAP1 and polymorphisms and urothelial cell carcinoma and can also provide effective information on the prevention of urothelial cell carcinoma.
